# Cucumber (*Cucumis sativus* L.) Leaf Extract as a Green Corrosion Inhibitor for Carbon Steel in Acidic Solution: Electrochemical, Functional and Molecular Analysis

**DOI:** 10.3390/molecules27123826

**Published:** 2022-06-14

**Authors:** Lijuan Feng, Shanshan Zhang, Long Hao, Hongchen Du, Rongkai Pan, Guofu Huang, Haijian Liu

**Affiliations:** 1 Shandong Engineering Research Center of Green and High-Value Marine Fine Chemical, Weifang University of Science and Technology, Weifang 262700, China; zhangshanshan87@wfust.edu.cn (S.Z.); duhongchen123@126.com (H.D.); panrk@wfust.edu.cn (R.P.); hgflh007@wfust.edu.cn (G.H.); liuhaijian.1987@163.com (H.L.); 2 CAS Key Laboratory of Nuclear Materials and Safety Assessment, Institute of Metal Research, Chinese Academy of Sciences, Shenyang 110016, China; chinahaolong@126.com

**Keywords:** carbon steel, extract, green corrosion inhibitor, quantum chemical calculation

## Abstract

An extract of cucumber leaves (ECSL) was prepared as a green corrosion inhibitor for carbon steel. Its carbon steel corrosion inhibition performance against 0.5 mol L^−1^ H_2_SO_4_ was investigated using electrochemical methods and scanning electron microscopy (SEM). Its composition was analyzed by gas chromatography and mass spectroscopy (GC−MS). Quantum chemical calculations and molecular dynamics simulations (MDS) were conducted to elucidate the adsorption mechanism of the inhibitor molecules on the carbon steel surface. The results indicated that the inhibition efficiency increases with its increasing concentration. The extract acted as a mixed type corrosion inhibitor, and its inhibition properties were ascribed to the geometric coverage effect induced by its adsorption on the metal surface in accordance with Langmuir’s law. The active components in the extract were identified as mainly organic compounds with functional groups such as aromatic moieties and heteroatoms. The inhibition activities of ECSL are delivered through the ability of the active components to adsorb on the metal surface through their functional groups to form a protective layer which hinders the contact of aggressive substances with carbon steel and thus suppresses its corrosion. This research provides an important reference for the design of green corrosion inhibitors based on plant waste materials.

## 1. Introduction

As one of the basic industrial materials, carbon steel has been widely applied in various fields due to its excellent mechanical properties and low cost [1,2,3,4,5,6,7,8]. Nevertheless, it is prone to suffer from scale and corrosion due to its chemical and electrochemical interactions with the surrounding environment—which leads to the degradation of metallic structures [3,9,10,11,12]. Every year, a large amount of carbon steel equipment is damaged and replaced owing to metal corrosion, causing enormous economic losses and huge potential safety risks [13]. The most widely applied method for removing scale during facility maintenance activities is pickling [14,15]. Sulfuric acid is one of the most commonly used pickling agents, as it can effectively remove dirt and oxidation products from the metal surface [15,16,17]. However, the corrosion rate of carbon steel is so high in acidic conditions that the equipment may be damaged during the acidic cleaning process. Therefore, applying corrosion inhibitors is considered to be an unavoidable approach, and has been proven to be a convenient, effective and economical technique for protecting metals during such processes [5,11]. It has been revealed that organic compounds containing heteroatoms, unsaturated bonds and planer conjugated systems usually display excellent corrosion inhibition activities because they adsorb onto the metal surface via their functional groups and form a protective film [3,5,18,19]. Unfortunately, traditional inhibitors are often toxic to human beings or have a detrimental impact on the environment, and so their use has been limited by more and more countries. Thus, researchers are keen to develop environmentally benign “green” corrosion inhibitors [3,10,11,12,13,14,20,21,22,23,24,25,26]. A perusal read of the literature has revealed that green inhibitors prepared from plant extracts are readily available, nontoxic, biodegradable and economical, and can be considered to be a most promising choice [16,23,24,25,26].

Cucumber (*Cucumis sativus* L.) is a vegetable that is widely planted in many countries. Its leaves are generally recognized as a biological waste material, causing serious ecological and environmental pollution. Meanwhile, it has been found that there are many valuable chemical constituents, including alkanoids, flavonoids, carbohydrates, proteins, sugars and steroids, in the leaves of plants [20,27,28,29,30]. These organic compounds contain adsorption centers—polar functional groups with N, S and O atoms or aromatic rings—in their molecular structures, which suggests that leaf extracts could be potential corrosion inhibitors [12,25,29,30,31,32]. However, there is no report to date on the corrosion inhibition performance of an extract based on cucumber leaves. Thus, if a corrosion inhibitor can be prepared from cucumber leaves, and if its corrosion inhibition mechanism can be determined, it will not only add a cheap new green product to the corrosion inhibitors list, but it will also provide a novel solution for the utilization of plant waste such as cucumber leaves. Therefore, in this study, an extract of cucumber leaves (ECSL) was prepared using an impregnation method, and its corrosion inhibition performance on carbon steel subjected to 0.5 mol L^−1^ H_2_SO_4_ solution was evaluated through electrochemical measurements and surface analysis techniques. The active components of the extract were analyzed using gas chromatography and mass spectrometry. The adsorption performance of the active constituents on the surface of carbon steel was further revealed through theoretical calculations and simulations. The aims of the work were to assess the corrosion inhibition effects of the biological waste product; to reveal its inhibition mechanisms; to pave a way for the application of low-cost green plant extracts; and to promote the realization of resources recycling.

## 2. Results

ECSL was prepared as described in “Materials and Methods”, Section 3. Based on the experimental and theoretical analysis methods described in Section 3, the results are shown and discussed as follows.

### 2.1. Potentiodynamic Polarization Curves

**Figure 1:**
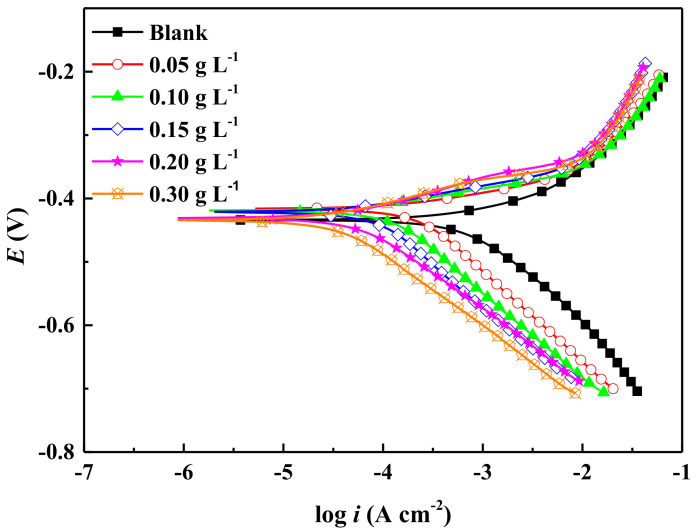
Potentiodynamic polarization curves of the carbon steel samples in 0.5 mol L^−2^ H_2_SO_4_ solution with and without different concentrations of ECSL.

Potentiodynamic polarization curves obtained from immersing the electrodes in 0.5 mol L^−1^ H_2_SO_4_ solution with and without various concentrations of ECSL are shown in Figure 1. Curve fitting results obtained by Tafel extrapolation are shown in Table 1, where *E*_corr_ denotes the free corrosion potential; *i*_corr_, the corrosion current density; *β*_c_, the cathodic Tafel slope; and *β*_a_, the anodic Tafel slope.

*IE* stands for the inhibition efficiency, which can be calculated according to the following equation:

*IE* (%) = (*i*^0^_corr_ − *i*_corr_)/*i*^0^_corr_ × 100% (1)

where *i*^0^_corr_ and *i*_corr_ are corrosion current densities for the uninhibited and inhibited samples, respectively [5].

It can be seen from Figure 1 and Table 1 that the addition of ECSL led to both the anodic and cathodic branches of the polarization curves moving toward lower current density directions, indicating that both the anodic and cathodic reactions were inhibited by ECSL [33,34]. Moreover, the corrosion current reduced with an increasing concentration of ECSL, suggesting that the more inhibitor molecules that were adsorbed on the sample surface, the more the contact between the metal surface and the corrosion medium was blocked. However, the changes in the anodic and cathodic Tafel slopes were very small, and the fluctuation of the corrosion potential was much less than 85 mV. Thus, it could be inferred that ECSL acted as a mixed type inhibitor which inhibited carbon steel corrosion by forming a protective structure, obstructing both the anodic and cathodic electrochemical reactions of the corrosion process [5,35,36].

### 2.2. Electrochemical Impedance Spectroscopy (EIS) Measurements

**Figure 2:**
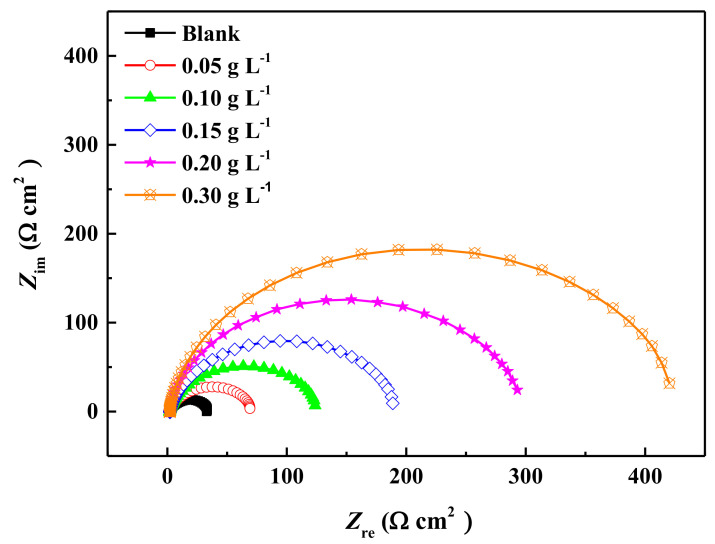
Electrochemical impedance spectrum of carbon steel samples in 0.5 mol L^−1^ H_2_SO_4_ solution with and without different concentrations of ECSL.

**Figure 3:**
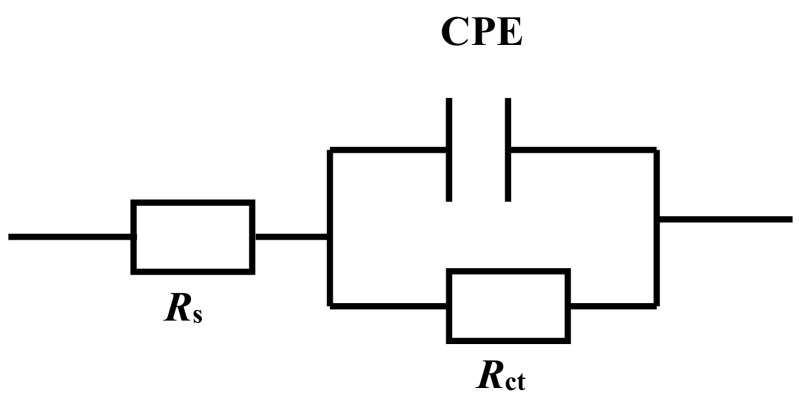
Equivalent circuit to fit the impedance data for carbon steel in 0.5 mol L^−1^ H_2_SO_4_ solution with and without different concentrations of ECSL.

Figure 2 shows the electrochemical impedance spectrum for carbon steel electrodes in 0.5 mol L^−1^ H_2_SO_4_ solution with and without various concentrations of ECSL. It is obvious that only one capacity loop appeared in the Nyqusit diagram for all electrodes, indicating that the carbon steel corrosion was mainly controlled by the charge transfer reaction [37]. However, the impedance behavior of the carbon steel sample was significantly changed by the presence of ECSL. It is notable that the radius of the capacitive loop enlarged with increasing ECSL concentration, suggesting that the anti-corrosion properties were gradually enhanced due to the increasing surface coverage of the electrode with increasing inhibitor concentration [33]. The EIS activities for the carbon steel samples in acidic solution, either with or without ECSL, can be interpreted using a model that describes a solution resistor element connecting with a unit of charge transfer resistor and double layer capacitor in parallel [*R*(*QR*) equivalent circuit], as shown in Figure 3; where *R*_s_ represents the solution resistance, *R*_ct_ the charge transfer resistance and *C*_f_ the capacitance of the double electric layer. Since all the capacitive semicircles were depressed (Figure 2), the constant phase element CPE (*Q*) was used to replace *C* for the description of the frequency independent phase shift induced by the roughness of the electrode surface [38,39]. The value of CPE can be assessed using the following equation:

*Z* (*ω*) = (*Z*_0_)^−1^ (*jω*) ^−^*^n^* (2)

where *Z*_0_ is the CPE constant, *ω* is the angular frequency (in rad/s), *j*^2^ = −1, is the imaginary number and *n* is the CPE exponent. Depending on *n*, CPE can represent resistance [*Z* (CPE) = *R*, *n* = 0], capacitance [*Z* (CPE) = *C*, *n* = 1], inductance [*Z* (CPE) = *L*, *n* = −1] or Warburg impedance (for *n* = 0.5). Thus, the inhibition efficiency can be calculated from the following formula:

*η* (%) = *θ* (%) = (1 − *R*_ct_/*R*^0^_ct_) × 100% (3)

where *η* denotes the inhibition efficiency and *θ* indicates the surface coverage [40,41,42]. The curve fitting results based on the *R*(*QR*) circuit are displayed in Table 2.

Based on Table 2, the capacity of the electrode was decreased in the presence of ECSL; this is ascribed to the fact that the acidic solution with its larger dielectric constant was replaced by the extract molecules with a smaller dielectric constant, which improved the double layer structure [43,44]. As the concentration of the inhibitor was increased, the double layer became thicker, reducing the local dielectric constant; which led to a decrease in the double layer capacity [45]. Meanwhile, the charge transfer resistance increased with increasing inhibitor concentration, indicating increasing mitigation of electrochemical corrosion. This is because the active molecules in ECSL were adsorbed onto the carbon steel surface and the charge transfer process was reduced, causing a corrosion inhibition effect.

### 2.3. Scanning Electron Microscopy (SEM) Observations

**Figure 4:**
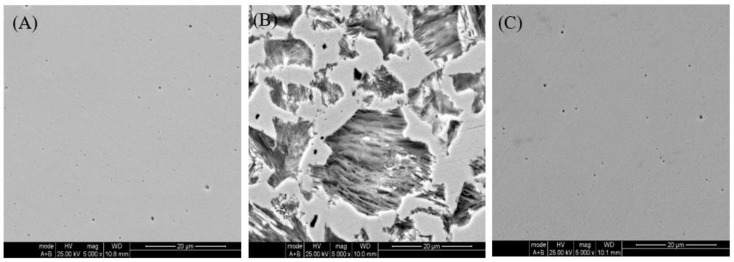
SEM images. (**A**) carbon steel sample before immersion; (**B**) carbon steel sample immersed in 0.5 mol L^−1^ H_2_SO_4_ solution; (**C**) carbon steel sample immersed in 0.5 mol L^−1^ H_2_SO_4_ solution with 0.20 g L^−1^ ECSL.

SEM observations were conducted with the aim of confirming the changes in the surface morphologies of carbon steel samples before and after the addition of ECSL. Figure 4 depicts the SEM images of samples immersed in 0.5 mol L^−1^ H_2_SO_4_ solution in the absence and presence of ECSL. For comparison purposes, an image of the morphology of the sample before immersion is also included. It is clear that many large holes appeared in the surface of the sample immersed in acid without ECSL, indicating that the surface of the carbon steel was seriously damaged and the sample suffered from serious corrosion. In the presence of ECSL, however, the surface of the sample was smooth, with only a few slight scratches induced by emery papers visible. It had an appearance similar to that of the sample before immersion, suggesting corrosion of the carbon steel was significantly inhibited by the plant extract. This might be ascribed to the active constituents of ECSL interacting with the metal surface and forming a protective layer, decreasing the contact area of the sample with the aggressive medium and consequently mitigating its corrosion [27,46].

### 2.4. Adsorption Isotherm

**Figure 5:**
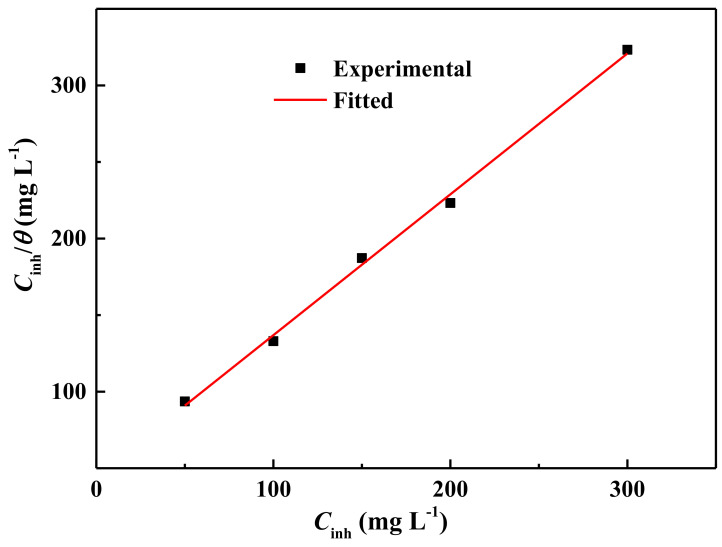
Langmuir adsorption isotherm for ECSL adsorbed on the carbon steel sample surface in 0.5 mol L^−1^ H_2_SO_4_ solution.

Previous researchers have confirmed that the adsorption behavior of organic molecules on metal surfaces has a decisive influence on their corrosion inhibition performance [47,48]. In order to further analyze the corrosion protection mechanism of ECSL, the surface coverage degree (*θ*) of different concentrations of the inhibitor was tested by fitting to several isotherms; the data fitted best with the Langmuir adsorption isotherm, as shown in Figure 5. According to Langmuir’s adsorption isotherm law, the relationship between *θ* and *C*_inh_ is shown by the following equation:

*C*_inh_/*θ* = 1/*K* + *C* (4)

where *K* represents the adsorption equilibrium constant, *C*_inh_ is the concentration of the extract, and *θ* is the surface coverage degree, which is approximately equal to *η* in Table 2 [5,49,50].

It is clear from Figure 5 that *C*_inh_/*θ* has a linear relationship with *C*_inh_, with a slope close to 1. The linear coefficient was 99.9% and the adsorption equilibrium constant *K* was calculated to be approximately 22.3 L g^−1^. This result indicated that the adsorption performance of ECSL on the carbon steel surface conformed to Langmuir’s adsorption law, and that the corrosion inhibition mechanism was the geometric covering effect.

### 2.5. Gas Chromatography and Mass Spectroscopy (GC–MS) Analysis

In order to further reveal the reasons for its corrosion inhibition effects, the constituents of ECSL were analyzed by GC−MS. The spectrum obtained is depicted in Figure S1. After preliminary analysis, it was identified that there are more than forty substances present in the extract, of which eleven compounds were most likely to have corrosion inhibition effects. These eleven chemical constituents, along with their molecular formula, abbreviations, retention time and molecular weight are listed in Table 3. As can be seen in Figure S1 and Table 3, it is notable that ECSL contains a variety of organic substances with multiple functional groups, such as rings, double bonds, π bonds and heteroatoms. Based on previous theories, such compounds have extraordinary adsorption potential, and are very promising corrosion inhibitors as they favor an ability to adsorb on the metal surface using these groups as adsorption centers, and subsequently inhibit metal corrosion.

### 2.6. Quantum Chemical Calculations

The corrosion activities of organic substances are closely related to their molecular, spatial and electronic structures. The adsorption of molecules mainly occurs relative to their frontier orbitals, namely, the highest occupied orbital (HOMO) and the lowest occupied orbital (LUMO). Further, their adsorption properties depend on the energy of the HOMO (*E*_HOMO_) and LUMO (*E*_LUMO_) [51,52,53]. The higher the *E*_HOMO_ of the molecule, the more difficult it is for the nucleus to attract electrons to its orbital, and therefore, the easier it is to donate electrons to form coordination bonds with the unoccupied orbital. The lower *E*_LUMO_ of the molecule, the easier it is to accept electrons and form feedback bonds. The difference in energy between the LUMO and HOMO (Δ*E* = *E*_LUMO_ − *E*_HOMO_) is also an important parameter to describe the reactivity of the inhibitor molecule toward the surface, if the difference in electronegativity between the molecule and the metal cannot be ignored [54]. In such cases, a higher Δ*E* suggests better stability of the molecule, and it will be more difficult for the molecule to participate in the adsorption reaction. In contrast, a lower Δ*E* indicates that it is easier to adsorb the molecule onto the metal surface to construct a protective film [21,55]. Generally, the effect on the molecular adsorption performance of a molecule at the metal surface is reflected in the parameter of global hardness (*H*).

**Figure 6:**
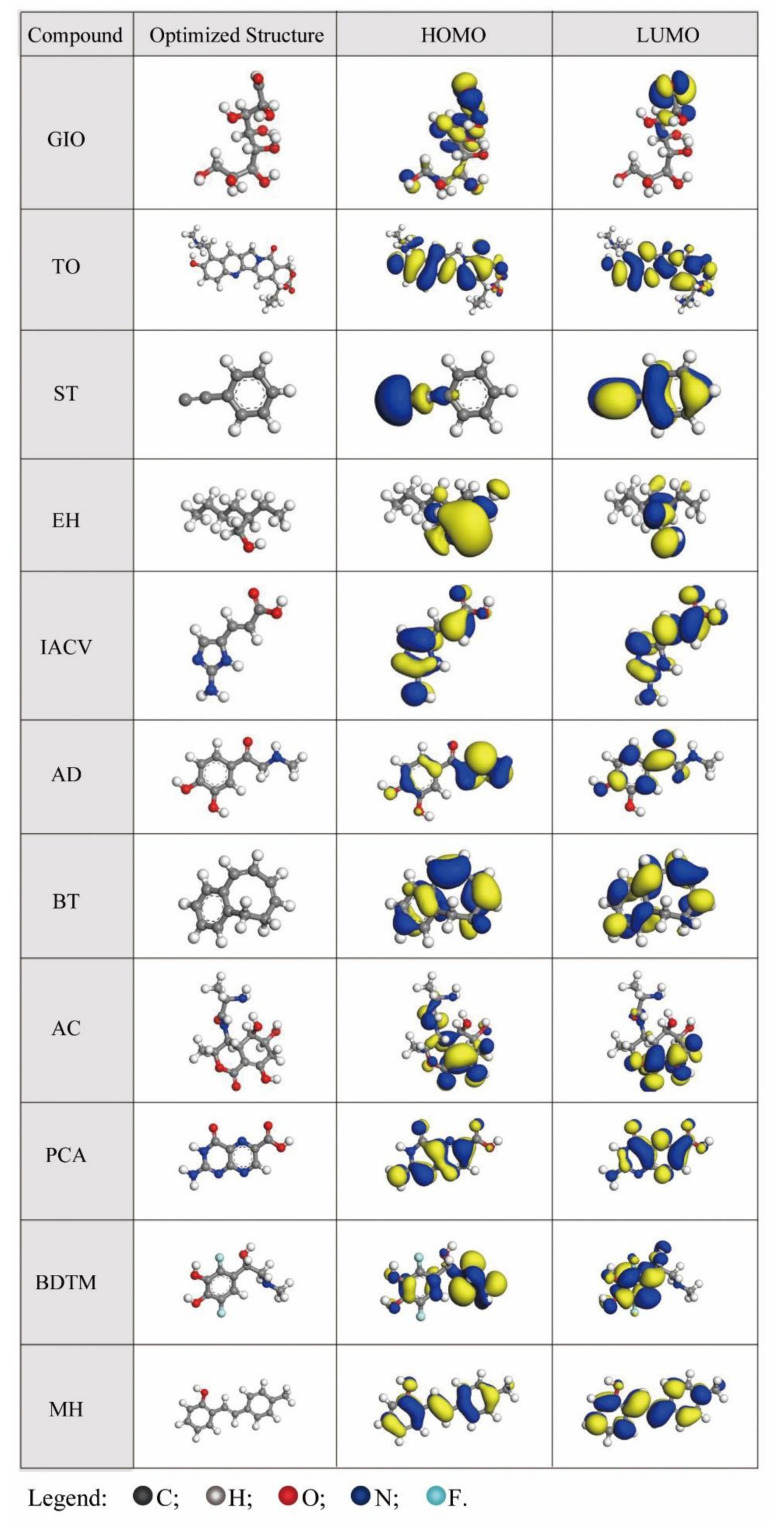
Optimized molecular structures and frontier molecular orbital density distributions of the eleven compounds identified in ECSL.

Figure 6 depicts the optimized molecular structures, and the HOMO and LUMO distributions of the eleven identified components. The locations of the HOMO and LUMO analyzed from the data in Figure 6 are shown in Table 4. Due to the density functional theory (DFT) not being suitable for computing the ionization potential and the electron affinity based on Koopman’s theorem [54,55,56], the Hartree–Fock (HF) method was subsequently used to calculate the values of *E*_LUMO_, *E*_HOMO_, Δ*E* and other quantum chemical parameters elucidated from *E*_LUMO_ and *E*_HOMO_.

According to Koopman’s theorem, the ionization potential (*I*) and the electron affinity (*A*) can be determined using the following equations, respectively:

*I* = −*E*_HOMO_ (5)

*A* = −*E*_LUMO_ (6)

The global hardness (*H*), the electronegativity (*X*) and the number of electrons transferred (Δ*N*) can be calculated according to the following relationships:

*H* = (*I* − *A*)/2 (7)

*X* = (*I* + *A*)/2 (8)

Δ*N* = (*X*_Fe_
*− X*_inh_)/2(*H*_Fe_
*− H*_inh_) (9)

where *X*_Fe_ = *I*_Fe_ = 7 eV, and *H*_Fe_ = 0 for iron, based on Pearson’s electronegativity scale assumption [57,58,59]. However, their application of *X*_Fe_ = 7 eV for the calculation of Δ*N* has been severely criticised because the value of 7 eV was obtained according to the free electron gas Fermi energy of iron using the free electron gas model. In that case, the electron–electron interaction is neglected, which causes differences from the state of the bulk metal, and therefore the use of this value is conceptually inappropriate. Thus, Kokalj suggested that the work function (*Φ*_Fe_) be used to replace *X*_Fe_. Hence, Δ*N* can be calculated as follows:

Δ*N* = (*Φ*_Fe_
*− X*_inh_)/2*H*_in_) (10)

Considering that metal corrosion most likely occurs at the densely packed surface, *Φ*Fe = 5.07 V obtained from the experimental result was applied to calculate Δ*N* [55,60]. The resulting structural parameters are listed in Table 5.

By carefully analyzing the data in Figure 6 and Table 4, we can elucidate that the HOMOs of the identified substances are mainly distributed in the aromatic rings (TO, AD, BT, BDTM, MH), heterocycles (TO, IACV, AC, PCA) and heteroatoms (GIO, TO, EH, IACV, AD, AC, BDTM, MH), which indicates that these sites have nucleophilic activities and are the preferred adsorption centers. Thus, the molecules can donate electrons to construct coordination bonds with iron and form a protective layer. The LUMOs of these substances are also mainly in benzene rings (TO, ST, AD, BT, BDTM, MH), heterocycles (TO, IACV, AC, PCA), and heteroatoms (GIO, TO, EH, IACV, AD, AC, PCA, BDTM, MH), which suggests that these compounds can accept electrons from the vacant d orbital of a metal to constitute a feedback bond. Therefore, it is possible that the molecules in ECSL first physically adsorb onto the surface of carbon steel, and then chemically interact with it to form a protective structure trough, sharing electron pairs between the ECSL molecules and the iron [61].

From Table 5, the difference in electronegativity between the molecule and the metal is visible in comparing the value of *X*_inh_ with that of *Φ*_Fe_; thus, the influence of Δ*E* on the molecular adsorption properties should be considered [54]. Consequently, the global hardness parameter (*H*), closely related to Δ*E*, indicates the charge transfer resistance, which is directly proportional to the energy change, including both the charge transfer to the molecule and the back donation from the molecule [62,63]. This indicates the reactivity of the inhibitor molecule towards the adsorption on a metallic surface. Generally, the reactivity of the molecule increases as the value of *H* or Δ*E* decreases. Thus, the inhibition efficiency of the molecule increases, since it is more possible to form a bond-anti-bond structure through offering and accepting electrons to and from the metal [21]. The results in Table 5 show that TO displays the lowest value, 4.63 eV, which is ascribed to the fact that this molecule contains the most rings and multiple heteroatoms. Most of the compounds have *H* values ranging from 5.0 to 6.1 eV. These values of *H* are a little higher than those calculated via DFT. This is reasonable since the *H* value calculated through DFT is lower than that found using the HF method, which has been confirmed by the studies of other researchers [57,64,65]. Accounting for the applicable condition of Koopman’s theorem, the *H* value obtained by the HF method is more reliable, and an *H* value in the range from 5.0 to 6.1 eV is suitable for a molecule with an inhibition effect [55,66,67]. In combination with Figure 6 and Table 4, these data indicate that these molecules all contain ring structures (benzene moiety or heterocyclic) with dislocation electrons. In terms of HSAB (hard/soft–acid/base) theory, these compounds can be recognized as soft bases which react with soft acids [68,69]. The *H* values of GIO and EH are the highest (6.85 and 7.53 eV, respectively) due to the fact that they only get negatively charged atoms and do not have conjugated structures. These two compounds can be recognized as hard bases. The inhibition effect of ECSL might be enhanced by the different types of compounds present. In the Fe–H_2_O system, there are different Lewis acids on the metal surface: the bare iron is categorized as a soft acid and the metal ions produced in the corrosion process are hard acids [70]. Soft bases such as TO can interact directly with the surface of the iron and form an adsorption film, protecting the metal from corrosion. EH and GIO, as hard bases, can interact with the Fe^3+^ or Fe^2+^ ions and assist the inhibition performance.

Electronegativity (*X*) reflects the ability of atoms to attract electrons toward themselves, and can be used to assess the tendency of a molecule to retain its own electrons during donor/acceptor interactions that lead to corrosion inhibition [59,62]. Usually, a compound with a lower value of *X* means that it is shares electrons with metals more easily, and can be expected to have higher inhibition performance [59,71]. From Table 5, there are four compounds (GIO, EH, TO and PCA) with an *X* value higher than 3.5 eV. Considering that GIO and EH are also the molecules with the highest *H* values, such high *X* values suggest they interact with the metal solely via a physical electrostatic effect, rather than by the chemical reaction of donating electrons. PCA and TO also have the lowest *E*_LUMO_, indicating that they are more likely to be electron acceptors rather than donors during their interaction with metals. The *X* values of the other seven substances have no significant differences, which may be ascribed to the fact that they all have planar ring structures with conjugated π bonds. Thereby, when they interact with metals, they are probably able to offer both electrons to the empty orbital of a metal molecule and receive electrons to form back-donation bonds, and as a result, they can strongly adsorb on the metal surface and constitute a protective layer, inhibiting its corrosion.

The fraction number of electrons transferred (Δ*N*) indicates inhibition efficiency resulting from electron donation [57]. If Δ*N >* 0, electrons can be transferred from organic molecules to metals and form feedback (back-donation) bonds. If Δ*N* < 3.6, the inhibition efficiency increases with increasing ability to donate electrons to the metal surface [61,72]. Per Table 5, the Δ*N* values of all the compounds are in the range from 0 to 3.6, revealing that they can interact with the metal via electron donation from the inhibitor to the metal surface.

### 2.7. Molecular Dynamics Simulations (MDS)

Molecular dynamics simulation is a comprehensive technology that combines mathematics, chemistry and physics through computational simulations to calculate the properties of materials in the system [61,71,72,73]. In order to better understand the interaction between ECSL and the carbon steel surface, and furthermore, to elucidate its corrosion inhibition mechanism, MDS was performed to analyze the adsorption behaviors of the active components of ECSL on the Fe(110) surface at the molecular level.

**Figure 7:**
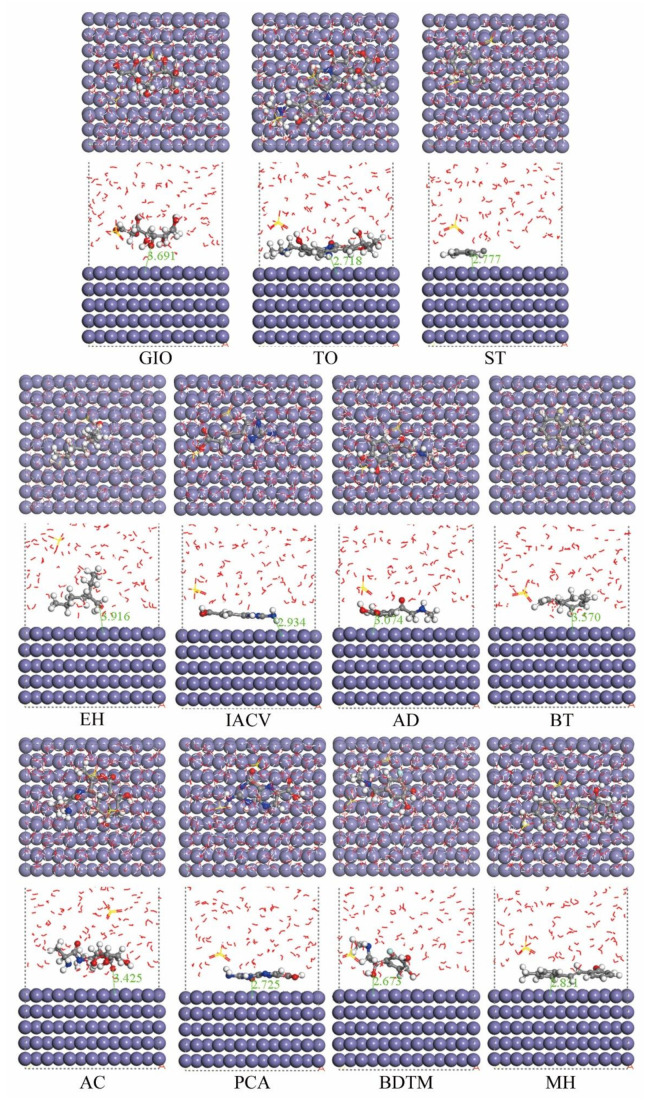
Optimized adsorption configurations of the molecular structures on Fe(110) surface.

The optimized adsorption configurations of the molecular structures on the metal surface are illustrated in Figure 7. The adsorption configuration of each molecule on the Fe(110) surface in 0.5 mol L^−1^ H_2_SO_4_ solution can be vividly observed by comprehensive analysis of Figure 6 and Figure 7, and Table 5. The GIO molecule adsorbs on the iron surface with the O atoms on its one side supporting like feet. TO adsorbs on the Fe(110) surface with the benzene rings and imidazole plane parallel to it. ST adsorbs with its benzene ring parallel to the Fe(110) surface and its hydrophobic carbon chain tilting upward. EH adsorbs using the O atom as the adsorption center with a C atom assisting. The adsorption centers of IACV are an imidazole ring as well as the O atoms; while those of AD are O atoms and the benzene ring. As to the adsorption of BT, the benzene ring is parallel to the Fe(110) surface, with the C atom supported on one side. AC adsorbs with the aromatic moieties parallel to the metal surface; meanwhile, the N and O atoms serve as adsorption centers to strengthen its adsorption. The PCA molecule horizontally adsorbs on the Fe(110) surface through the double ring structure, while the O and N atoms of the branched chain can also perform as active adsorption centers, which facilitates its adsorption. Due to space configuration constraints, BDTM adsorbs on the metal surface at a certain angle, yet its heteroatoms can still act as the adsorption centers. MH molecules adsorb mainly through their benzene rings.

In general, by careful examination of Figure 7, it can be noted that all eleven compounds identified in ECSL are able to adsorb on the Fe(110) surface, therefore a barrier structure can be formed to isolate contact between the carbon steel surface and the corrosive medium, thus mitigating the corrosion of the carbon steel.

It should be mentioned that, owing to spatial structure limitations, the adsorption configuration of an organic molecule on a metal surface is not the same as the optimal geometric structure obtained by quantum chemical calculations; instead, it is adjusted to a certain extent. As a result, the distance from the metal surface to the adsorbed molecules varies. As shown in Figure 7, for molecules with less active groups, like EH and GIO, the distance between the molecule and the metal surface is obviously larger than that of other molecules. In fact, the high values (3.916 and 3.916 Å, respectively) indicate their interactions with the metal are through physical adsorption mechanisms rather than chemical ones [55]. Compounds containing planer moieties, especially benzene rings, interact easily with the metal and can adsorb onto the Fe(110) surface at a short distance, since the planer structure can be parallel to the Fe(110) surface. ST, IACV, AD, PCA, BDTM and MH all display such adsorption configurations. However, if the branched chains in the molecule are too many or too long, and they cannot adjust to the same side of the planar structure, such as in BT and AC, the molecule will not adsorb on the metal surface so closely. These results are expected to provide a reference for the design of new high-efficiency corrosion inhibitors.

The adsorption energy (*E*_ads_) between the iron surface and the organic compound can be calculated as follows:

*E*_ads_ = *E*_total_ − (*E*_surf+solu_+ *E*_inh_) (11)

where *E*_total_ is the total energy of the inhibitor molecule adsorption system on the Fe(110) surface in 0.5 mol L^−1^ H_2_SO_4_ solution, and *E*_surf+solu_ + *E*_inh_ is the sum of the energy including the metal surface, the corrosive solution and the inhibitor molecule before adsorption [72,74]. The calculated results are listed in Table 6 from the lowest to the highest adsorption energy value. It has been acknowledged that a negative adsorption energy value indicates spontaneous adsorption of a molecule on a metal surface. Moreover, a lower value of *E*_ads_ implies the molecule is more likely to adsorb on the metal surface, thus, it exhibits a better corrosion inhibition performance [58,59]. It can be observed from Table 6 that the adsorption energy on the Fe(110) surface for each molecule has a negative value, indicating that all eleven components identified in ECSL are able to adsorb on the carbon steel surface and exhibit corrosion inhibition effects.

### 2.8. Corrosion Inhibition Mechanism

The corrosion inhibition mechanism of ECSL for carbon steel can be analyzed from the chemical structures of the identified components. The main chemical constituents of ECSL are organic compounds containing many functional groups, such as benzene rings, heterocycles and polar atoms, which have delocalized or unpaired electrons and demonstrate high *E*_HOMO_. They are prone to provide electrons to the empty d orbital of Fe and adsorb on its surface. Meanwhile, by virtue of these functional groups, they have low *E*_LUMO_ and easily accept electrons from the d orbital of Fe to form feedback bonds and enhance the adsorption capacity. Thereby, the active components in ECSL can adsorb on the metal surface and constitute a protective structure. Moreover, ECSL contains a variety of active components, including both soft bases and hard bases, which can either directly adsorb on the carbon steel surface or interact with Fe^2+^ and/or Fe^3+^ to form a protective layer. Therefore, they complement each other, to a certain extent, to enhance the metal corrosion inhibition properties. As a result, ECSL can spontaneously and effectively adsorb on the carbon steel surface and inhibit its corrosion.

## 3. Materials and Methods

### 3.1. Materials

The metal samples were prepared from R235 carbon steel, which was machine cut into cuboid samples with the dimensions of 10 mm × 10 mm × 5 mm. The composition of the carbon steel is listed in Table 7. The sample was embedded in a PVC holder using epoxy resin, leaving only a working surface with an area of 1 cm^2^ for electrochemical measurements. Prior to each experiment, the working surface was abraded with sandpaper from 100 to 1000 mesh, degreased with acetone, rinsed in distilled water, and air-dried. The corrosive medium was 0.5 mol L^−1^ H_2_SO_4_ solution which was prepared from analytical reagent grade H_2_SO_4_ and bi-distilled water.

### 3.2. ECSL Preparation

Fresh cucumber leaves (collected from the green house of the agricultural experimental base at Weifang University of Science and Technology, China) were cleaned under running water and then distilled water to remove the surface dirt. Next, the leaves were dried in an oven at 45 °C for approximately 24 h. The dried leaves were then ground to a powder and screened with a sieve with the pore size of 0.15 mm. After that, the dried and powdered cucumber leaves were soaked in ethanol (75% by volume) for 2 h at 25 °C. Then, the plant extract was boiled at 45 °C, naturally cooled to room temperature, and triple-filtered. The excess ethanol was removed by vacuum distillation. Finally, the plant extract, a dark brown solid residue, was obtained.

### 3.3. Chemical Composition Analysis

In order to identify the effective components of the plant extract, GC−MS analysis was carried out using Agilent Technologies GC model 7890A and Mass Spectrometry model 5977, coupled with an HP-5 capillary fused silica column of dimensions 30 μm × 320 μm × 0.25 μm. The carrier gas was helium, the pressure was controlled at 11.604 Psi and the flow rate was 1.5 mL min^−1^. The oven temperature was programmed as follows: the initial temperature was set as 35 °C (isothermal for 5 min); then the temperature was increased to 90 °C at the rate of 6 °C min^−1^ (isothermal for 3 min); then to 150 °C at 5 °C min^−1^ (isothermal for 2 min); and finally, to 200 °C at 5 °C min^−1^ (isothermal for 3 min). All peaks were analyzed by matching with those in the NIST library to obtain the exact information.

### 3.4. Electrochemical Techniques

Electrochemical measurements were conducted using an electrochemical work station (CHI-660E, Chenhua) equipped with a three-electrode system, where the carbon steel sample served as the working electrode, a platinum plate as the auxiliary electrode and a saturated calomel electrode (SCE) as the reference electrode. The polarization curves were determined from a cathodic potential of −0.25 V to an anodic potential +0.25 V, with respect to the open circuit potential (OCP), at a sweep rate of 1 mV s^−1^. Electrochemical impedance spectroscopy (EIS) plots were acquired in the frequency range from 100 kHz to 10 mHz with a perturbation amplitude of 10 mV. The EIS spectra were fitted using the Corrview software. A fresh working electrode was used for each measurement. At least three runs were performed for each measurement to obtain reproducible data.

### 3.5. Surface Morphological Observation

The surface morphologies of the carbon steel samples immersed in 0.5 mol L^−1^ H_2_SO_4_ solution for 2 h with and/or without 0.20 g L^−1^ ECSL were observed by scanning electron microscopy (SEM, Philip XL 30) at 5000× magnification. The energy of the acceleration beam employed was 25 kV.

### 3.6. Computational Details

All of the quantum chemical calculations and molecular dynamics simulations were performed using Materials Studio 2019 software supplied by Biovia Community. The molecular structures of the inhibitors were fully geometrically optimized by DMol3 module using the function of PBE (proposed by Perdew–Burke–Ernzerhof) with the double numeric basis set of DNP in the DFT framework. The convergence tolerances for energy, maximum force and maximum atomic displacement were set as 1.0 × 10^−5^ Ha, 0.002 Ha Å^−1^ and 0.005 Å, respectively. The solvent effect was addressed using the COSMO implicit model of water. Since the Koopmans’ approximation may lose its validity under the DFT framework, the HF method was applied to calculate the structural parameters using cc-pVTZ by virtue of the Gaussian 09W version D.01 software. The adsorption behavior of each compound on the metal surface was investigated using the Forcite module with MDS. The simulations of the interactions of the molecules with the Fe(110) surface were conducted in periodic boxes with the dimensions of 17.2 Å × 16.2 Å × 75.5 Å using two layers. The bottom Fe(110) surface was fabricated with a five-layer slab model and the top solvent layer was constructed with 222 H_2_O, 1 organic molecule, 4 H^+^ and 2 SO_4_^2−^, where a 30 Å vacuum region was set to ensure the decoupling of the repeated slabs. The MDS were carried out under the conditions of 298 K, NVT ensemble, with a time step of 0.1 fs and simulation time of 500 ps.

## 4. Conclusions

A green corrosion inhibitor, ECSL, can be prepared from a plant waste, namely, cucumber leaves. It exhibits an excellent inhibition effect on the corrosion of carbon steel in 0.5 mol L^−1^ H_2_SO_4_ solution, and its inhibition efficiency increases with increasing concentration. ECSL reduces both the cathodic and anodic reactions of carbon steel in H_2_SO_4_ solution. It is a mixed type corrosion inhibitor and its corrosion inhibition activities are attributed to the geometric coverage effect. Its interaction with the carbon steel surface is ascribed to spontaneous physical and chemical adsorption, which obeys the Langmuir adsorption law. The corrosion inhibition properties of ECSL are closely related to the unique molecular structures of its active components with many functional groups. The molecules can easily adsorb on the carbon steel surface using these functional groups as adsorption centers to form a protective layer, obstructing the corrosion medium and suppressing the corrosion of the carbon steel.

## Figures and Tables

**Table 1:** Curve fitting results for the potentiodynamic polarization curves for carbon steel in 0.5 mol L^−1^ H_2_SO_4_ solution with and without different concentrations of ECSL.

*C*_inh_ (g L^−1^)	*β*_a_ (mV dec^−1^)	*β*_c_ (mV dec^−1^)	*i*_corr_ (mA cm^−2^)	*E*_corr_ (mV)	*IE* (%)
0	58	125	0.73	−430	/
0.05	59	127	0.35	−420	51.7
0.10	59	126	0.19	−419	73.8
0.15	60	127	0.13	−422	82.1
0.20	61	127	0.066	−431	90.9
0.30	62	130	0.047	−434	93.5

**Table 2:** Curve fitting results of the electrochemical impedance data for carbon steel in 0.5 mol L^−1^ H_2_SO_4_ solution with and without different concentrations of ECSL.

*C*_inh_ (g L^−1^)	*R*_s_ (Ω cm^2^)	CPE−*C* (μF cm^−2^)	CPE−n	*R*_ct_ (Ω cm^2^)	*η* (%)
0	1.94	104	0.95	29.3	/
0.05	2.22	86.6	0.94	62.9	53.4
0.10	2.19	62.2	0.94	118	75.2
0.15	1.97	51.8	0.94	147	80.1
0.20	2.11	43.6	0.94	281	89.6
0.30	2.01	33.9	0.94	406	92.8

**Table 3:** Compounds identified from the GC−MS chromatogram, and molecular information assigned to the respective signals.

Name of the Compound	Abbreviation	Retention Time (min)	Molecular Formula	Molecular Weight
1-gala-l-ido-octose	GIO	1.737	C_8_H_16_O_8_	240
Topotecan	TO	2.215	C_23_H_23_N_3_O_5_	421
Styrene	ST	8.616	C_8_H_8_	104
2-ethyl-1-hexanol	EH	13.14	C_8_H_18_O	130
2-amino-5-[(2-carboxy)vinyl]-imidazole	IACV	15.24	C_6_H_7_N_3_O_2_	153
Adrenalone	AD	19.62	C_9_H_11_NO_3_	181
Benzocycloheptatriene	BT	22.02	C_11_H_10_	142
Actinobolin	AC	24.73	C_13_H_20_N_2_O_6_	300
Pterin-6-carboxylic acid	PCA	27.37	C_7_H_5_N_5_O_3_	207
2,5-difluoro-β, 3, 4-trihydroxy-N-methyl-benzeneethanamine	BDTM	30.74	C_8_H_11_F_2_N	219
4′-methyl-2-hydroxystilbene	MH	33.26	C_15_H_14_O	210

**Table 4:** HOMO and LUMO distributions of compounds identified in ECSL.

Compound	HOMO Distribution	LUMO Distribution
GIO	O	O
TO	Rings	Rings
ST	C in the branch	Rings
EH	Branch with O atom	Branch with O atom
IACV	Pentatomic ring, O, N	Pentatomic ring, O, N
AD	Benzene ring, N, O	Benzene ring, N, O
BT	Rings	Rings
AC	Rings, N	Rings, N
PCA	Rings, O	Rings, O
BDTM	O, N, Benzene ring	O, Benzene ring
MH	Benzene rings	Benzene rings

**Table 5:** Quantum chemical parameters of active components identified in ECSL.

Compound	*E*_HOMO_ (eV)	*E*_LUMO_ (eV)	ΔE (eV)	*H* (eV)	*X* (eV)	Δ*N*
GIO	−11.03	2.66	13.69	6.85	4.18	0.07
TO	−8.17	1.09	9.25	4.63	3.54	0.17
ST	−8.39	2.65	11.04	5.52	2.87	0.20
EH	−11.33	3.74	15.07	7.53	3.79	0.08
IACV	−7.80	2.35	10.14	5.07	2.72	0.23
AD	−8.64	2.42	11.05	5.53	3.11	0.18
BT	−8.09	2.60	10.69	5.35	2.75	0.22
AC	−9.09	2.75	11.83	5.92	3.17	0.16
PCA	−9.14	1.55	10.69	5.34	3.80	0.12
BDTM	−8.64	3.58	12.21	6.10	2.53	0.21
MH	−7.82	2.21	10.03	5.01	2.81	0.23

**Table 6:** Interaction energies for each component identified in ECSL with Fe(110) surface.

Compound	*E*_surf+solu_ + *E*_inh_ (kcal mol^−1^)	*E*_total_ (kcal mol^−1^)	*E*_ads_ (kcal mol^−1^)
TO	−30,302.11	−31,204.32	−902.21
PCA	−30,444.21	−31,331.86	−887.65
BDTM	−30,397.84	−31,277.37	−879.53
AC	−30,430.67	−31,246.72	−816.05
AD	−30,390.48	−31,200.68	−810.19
ST	−30,001.78	−30,759.53	−757.75
IACV	−30,570.80	−31,195.94	−625.14
MH	−30,411.15	−31,000.90	−589.75
BT	−30,353.40	−30,924.26	−570.86
GIO	−30,351.34	−30,850.19	−498.85
EH	−30,431.33	−30,870.30	−438.97

**Table 7:** Composition of carbon steel in wt. (%).

Composition	C	Si	Mn	P	S	Fe
Amount (%)	0.16	0.14	0.48	0.03	0.03	99.16

## Data Availability

Not Applicable.

## Supplementary Materials

The following supporting information can be downloaded at: https://www.mdpi.com/article/10.3390/molecules27123826/s1, Figure S1: GC−MS chromatogram of ECSL.

## Sample Availability

Samples of ECSL is available from the authors.
