# Supplementary material for: Cucumber (Cucumis sativus L.) Leaf Extract as a Green Corrosion Inhibitor for Carbon Steel in Acidic Solution: Electrochemical, Functional and Molecular Analysis
Source: Molecules. 2022 Jun 14;27(12):3826. doi: 10.3390/molecules27123826 (PMC9227098; doi:10.3390/molecules27123826)
Supplement: Supplementary file 1 [file molecules-27-03826-s001.zip › molecules-1700942-supplementary.pdf]

Cucumber (*Cucumis Sativus L.*) Leaf Extract as a Green Corrosion Inhibitor for Carbon Steel in Acidic Solution: Electrochemical, Functional and Molecular Analysis

Lijuan Feng [1,*], Shanshan Zhang [1], Long Hao [2], HongChen Du [1], Rongkai Pan [1], Guofu Huang [1] and Haijian Liu [1]

[1] Shandong Engineering Research Center of Green and High-Value Marine Fine Chemical, Weifang University of Science and Technology, Weifang, Shandong 262700, China; lgylgy@wfust.edu.cn
[2] CAS Key Laboratory of Nuclear Materials and Safety Assessment, Institute of Metal Research, Chinese Academy of Sciences, Shenyang, Liaoning 110016, China; chinahaolong@126.com
* Correspondence: ljfeng@alum.imr.ac.cn; Tel.: +86-0536-5107-638

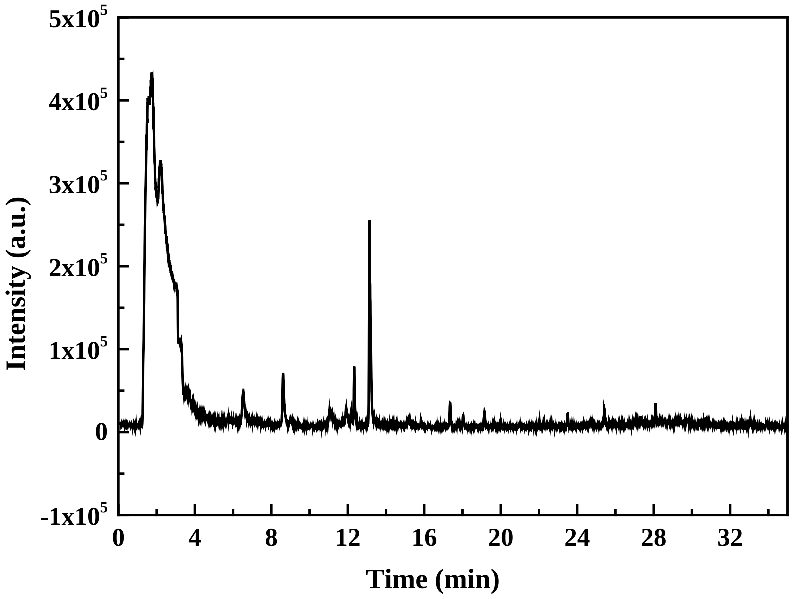

Figure S1. GC–MS chromatogram of ECSL.
